# Time From Diagnosis of Lewy Body Dementia to Death: Retrospective Study Exploring Patients Within Humber Older People's Mental Health Services

**DOI:** 10.1192/bjo.2024.457

**Published:** 2024-08-01

**Authors:** Sunday Adeoye, Manorama Bhattarai

**Affiliations:** Humber NHS Trust, Hull, United Kingdom

## Abstract

**Aims:**

Lewy Body Dementia (LBD) is the second commonest dementia. It accounts for around 7% of dementia cases in secondary care. Studies have shown that LBD patients have an accelerated trajectory towards death when compared with other forms of dementia. Studies have suggested that LBD cases, as compared with Alzheimer dementia, have accelerated cognitive decline, more comorbid conditions, a higher mortality rate, greater service use and poorer quality of life. Most previous studies of LBD have been based on select research cohorts, so less is known about the naturalistic patterns, characteristics, and outcomes of the disease in routine clinical settings.

The aim of the study is to determine the average duration from the time of diagnosis to death among patients with Lewy body dementia in OPMH to understand the prognostic pattern of LBD in our locality.

Objectives

1. To determine the commonest age of diagnosis and death of patients diagnosed with LBD in OPMH.
2. To explore sociodemographic distribution of patients within the study population.
3. To determine the time from diagnosis to death of patients diagnosed with LBD in OPHM.
4. To determine the common psychotropics combinations used in management of LBD in our psychogeriatric unit.

**Methods:**

This is a retrospective cross-sectional study of all the patients with diagnosis of LBD that presented to Humber Older People Mental Health Services in Hull. The sample consisted of electronic records of all 39 patients under the team but only 38 met the inclusion criteria. Patients’ records were reviewed and information such as gender, ethnicity, age at diagnosis, age at death or age at recruitment if alive, and psychotropic medication they are/were on was retrieved from the records. The time from diagnosis to death was obtained by subtracting age at diagnosis from age at death and this is recorded in years.

**Results:**

The result showed that majority of our patients were male and about 68.4% of our patients received their diagnosis between the age of 70 and 84 years and that 59.3% of them died within 5 years of receiving their diagnosis. The result also showed that the commonest psychotropic prescribed for LBD patients were single anticholinesterase inhibitor (donepezil or rivastigmine).

**Conclusion:**

This study showed that majority of patients died within 5 years of receiving their diagnosis of Lewy body dementia. This underscores the fatality and mortality associated with Lewy body dementia. More needs to be done in developing strategies to ensure improved awareness of Lewy body dementia in our community.

